# Spindle Assembly Checkpoint Protein Dynamics Reveal Conserved and Unsuspected Roles in Plant Cell Division

**DOI:** 10.1371/journal.pone.0006757

**Published:** 2009-08-27

**Authors:** Marie-Cécile Caillaud, Laetitia Paganelli, Philippe Lecomte, Laurent Deslandes, Michaël Quentin, Yann Pecrix, Manuel Le Bris, Nicolas Marfaing, Pierre Abad, Bruno Favery

**Affiliations:** 1 Institut National de la Recherche Agronomique, Unité Mixte de Recherche 1301, Sophia-Antipolis, France; 2 Centre Nationale de la Recherche Scientifique, Unité Mixte de Recherche 6243, Sophia-Antipolis, France; 3 Université de Nice Sophia-Antipolis, Unité Mixte de Recherche 1301, Sophia-Antipolis, France; 4 Institut National de la Recherche Agronomique-Centre National de la Recherche Scientifique, Unité Mixte de Recherche 2594, Castanet-Tolosan, France; 5 Institut Méditerranéen d'Ecologie et de Paléoécologie IMEP, Unité Mixte de Recherche- Centre National de la Recherche Scientifique –Institut de Recherche pour le Développement 6116, Université Paul Cézanne, Marseille, France; Oregon State University, United States of America

## Abstract

**Background:**

In eukaryotes, the spindle assembly checkpoint (SAC) ensures that chromosomes undergoing mitosis do not segregate until they are properly attached to the microtubules of the spindle.

**Methodology/Principal Findings:**

We investigated the mechanism underlying this surveillance mechanism in plants, by characterising the orthogolous SAC proteins BUBR1, BUB3 and MAD2 from *Arabidopsis*. We showed that the cell cycle-regulated BUBR1, BUB3.1 and MAD2 proteins interacted physically with each other. Furthermore, BUBR1 and MAD2 interacted specifically at chromocenters. Following SAC activation by global defects in spindle assembly, these three interacting partners localised to unattached kinetochores. In addition, in cases of ‘wait anaphase’, plant SAC proteins were associated with both kinetochores and kinetochore microtubules. Unexpectedly, BUB3.1 was also found in the phragmoplast midline during the final step of cell division in plants.

**Conclusions/Significance:**

We conclude that plant BUBR1, BUB3.1 and MAD2 proteins may have the SAC protein functions conserved from yeast to humans. The association of BUB3.1 with both unattached kinetochore and phragmoplast suggests that in plant, BUB3.1 may have other roles beyond the spindle assembly checkpoint itself. Finally, this study of the SAC dynamics pinpoints uncharacterised roles of this surveillance mechanism in plant cell division.

## Introduction

In eukaryotes, the spindle assembly checkpoint (SAC) is a sophisticated surveillance mechanism that ensures the fidelity of chromosome segregation during mitosis [1], [2]. The SAC monitors the interaction between chromosomes and microtubules (MTs) at specialised chromosomal regions, the kinetochores. In response to unattached kinetochores and to kinetochores lacking tension, the SAC is activated and localised to unattached kinetochores. The SAC transmits a “wait anaphase” signal until all chromosomes achieve bipolar attachment. This signal is transmitted through the inhibition of anaphase-promoting complex/cyclosome (APC/C) activity by sequestration of the CDC20 cofactor. SAC components were first identified through genetic screens in budding yeast and include the MAD (mitotic arrest-deficient) and BUB (budding uninhibited by benzymidazol) proteins [3], [4]. In metazoans and yeast, the mitotic checkpoint complex (MCC), which contains the three SAC proteins MAD2, MAD3 (equivalent of BUBR1, for BUB1-related, in higher eukaryotes) and BUB3 together with CDC20, is regarded as the SAC effector [5]–[7]. In budding yeast, the SAC is a non-essential device and it only becomes essential in response to ‘damage’ that is perturbations in the kinetochore-MT attachment process [3], [4]. On the other hand, in metazoans, the SAC is an essential pathway, the integrity of which is required to prevent chromosome mis-segregation and cell death [2]. In plants, SAC protein homologs have been identified *in silico* [8]–[10], but function has been investigated only for MAD2 for which localisation to unattached kinetochores has been demonstrated by immunolocalisation [11], [12].

In this paper, we investigated how this surveillance mechanism operates in the green kingdom. We demonstrated physical interactions between *A. thaliana* BUBR1, BUB3.1 and MAD2 and their dynamics at unattached kinetochores. In cases of ‘wait anaphase’, plant BUBR1, BUB3.1 and MAD2 proteins were unexpectedly associated with both kinetochores and kinetochore microtubules. Our findings suggest that plant BUBR1, BUB3.1 and MAD2 have both the SAC protein functions conserved from yeast to humans and pinpoints uncharacterised roles in plant cell division.

## Results and Discussion

As a first attempt to study SAC during the plant cell cycle, candidate *A. thaliana* orthologs of the human essential mitotic checkpoint complex proteins BUBR1, BUB3 and MAD2 were identified by OrthoMCL [13] clustering of orthologous proteins from six model eukaryotic species. The six complete proteomes compared included those of plants (*A. thaliana* and *Oryza sativa*), human (*Homo sapiens*), insect (*Drosophila melanogaster*) and nematodes (*Caenorhabditis elegans* and *Meloidogyne incognita*). *A. thaliana* BUBR1 (AT2G33560) is a 46 kD protein containing an N-terminal MAD3-BUB1 conserved domain and two KEN boxes conferring substrate recognition by APC/C [14] (Fig. S1). These two KEN boxes are conserved from yeast MAD3 to human BUBR1 and are required for the concerted action of MAD3 and MAD2 in the checkpoint inhibition of CDC20-APC/C [15]–[17]. Like the MAD3 proteins of *Saccharomyces cerevisiae* and *Schizosaccharomyces pombe*, *A. thaliana* BUBR1 differs from human BUBR1 by the absence of a C-terminal kinase domain. However, the kinase activity of BUBR1 has been shown to be dispensable for spindle checkpoint function in *Xenopus larvei* [18]. Two *A. thaliana* BUB3 proteins (BUB3.1, AT3G19590; BUB3.2, AT1G49910) were identified. Both are 38 kD proteins containing WD40 repeats, which have been shown to be involved in the association of BUB3 with MAD2, MAD3 and CDC20 in yeast [19]. *A. thaliana* BUB3.1 and BUB3.2 are 88% identical. BUB3.1 is 52% and 22% identical to the human and *S. cerevisiae* BUB3 proteins [4], [20], respectively, over its entire length (Fig. S2). *A. thaliana* MAD2 (AT3G25980) is a 24 kD protein containing a HORMA domain. It is 44% identical to the human MAD2 protein [21] and 81% identical to the maize MAD2 protein [11], over its entire length (Fig. S3).

### Arabidopsis *BUBR*, *BUB3.1 and MAD2* genes were expressed in tissues enriched in dividing cells

**Figure 1:**
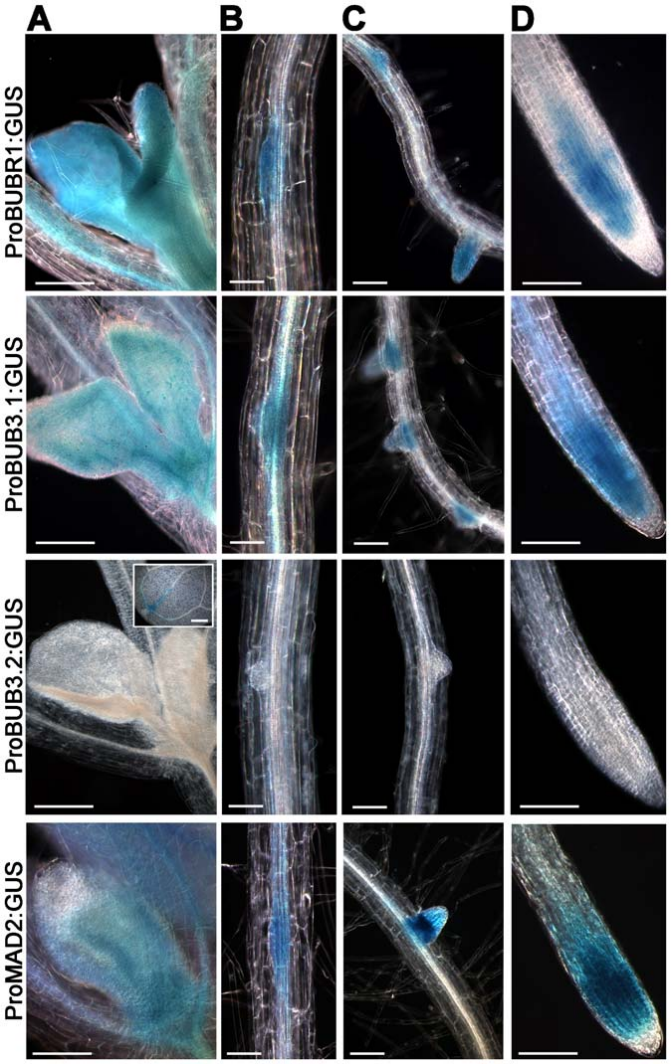
The pattern of expression of *BUBR1*, *BUB3.1*, *BUB3.2* and *MAD2* during *A. thaliana* development. Promoter:GUS fusions revealed *BUBR1*, *BUB3.1* and *MAD2* expression in the developing leaves of 7-day-old seedlings (A), in lateral root primordia (B), lateral root meristems (C) and root meristems (D). *BUB3.2* expression was only detected in cotyledons (insert). Bars, 200 µm (A), 50 µm (B), 100 µm (C and D).

We investigated the pattern of expression of the *A. thaliana BUBR1, BUB3.1, BUB3.2* and *MAD2* genes during plant development, using *A. thaliana* transgenic lines transformed with the corresponding promoter-GUS reporter gene constructs. Similar patterns of GUS expression were observed for the *BUBR1*, *BUB3.1* and *MAD2* promoters, both of which directed expression in tissues with a high proportion of dividing cells, early in organ development, in young leaves (Fig. 1A), lateral root primordia (Fig. 1B), lateral root meristems (Fig. 1C) and root meristems (Fig. 1D). Individual cells with strong GUS activity were observed in root meristems. In contrast to the cell cycle regulated pattern observed for both *BUBR1*, *BUB3.1* and *MAD2* promoter, no GUS activity in dividing cells was observed for the *BUB3.2* promoter in young leaves (Fig. 1A), lateral root primordia (Fig. 1B), lateral root meristems (Fig. 1C) and root meristems (Fig. 1D). These results are consistent with global transcriptome and RT-PCR analysis showing that *BUB3.1*, *BUBR1* and *MAD2* presented a distinct expression peak at the G2/M boundary in synchronised *A. thaliana* cell cultures that was not observed for *BUB3.2* [9], [10]. Because *BUB3.2* was not a cell cycle regulated gene, we next focused on *BUB3.1* candidate gene.

### BUBR1 and MAD2 interact specifically at chromocenters

**Figure 2:**
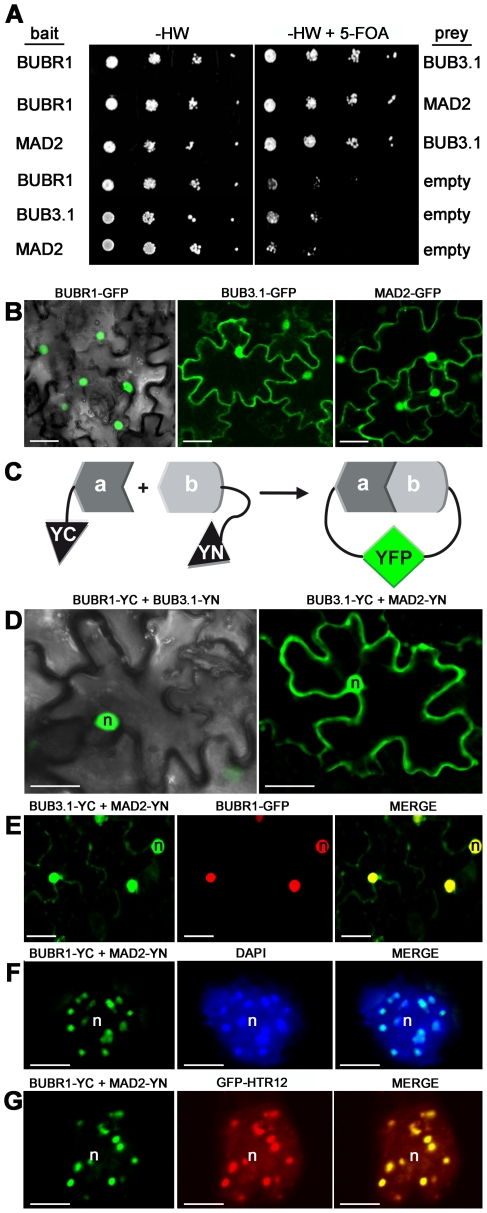
Interactions between *Arabidopsis* BUBR1, BUB3.1 and MAD2 in yeast and *in planta*. (A) Interactions in the yeast two-hybrid split-ubiquitin system. Dilution series of yeast JD53 cells expressing both bait fusions (BUBR1 or MAD2:Cub:URA3) and prey fusions (Nub:BUB3.1 or MAD2) were grown on yeast medium minus histidine and tryptophan (-HW) but containing 5-FOA, as indicated. Interaction resulted in uracil auxotrophy and 5-FOA resistance. (B) Single-plane images of tobacco epidermal leaf cells infiltrated with *A. tumefaciens* expressing BUBR1:GFP, BUB3.1:GFP or MAD2:GFP constructs. (C) Principle of *in vivo* bimolecular fluorescence complementation (BiFC). BiFC is based on the fusion of the prey (a) and bait (b) to the N- and C-terminal halves of the yellow fluorescent protein YFP (YN and YC, respectively), forming a functional YFP upon interaction [23]. (D–G) *In planta* BiFC assay. Single-plane confocal images of epidermal leaf cells infiltrated with *A. tumefaciens* co-expressing (D) BUBR1-YC and BUB3.1-YN or BUB3.1-YC and MAD2-YN, (E) BUB3.1-YC and MAD2-YN (green channel) and BUBR1-GFP (red channel), (F) BUBR1-YC and MAD2-YN, (G) BUBR1-YC and MAD2-YN (green channel) and GFP-HTR12 (red channel) fusion constructs. The merged images show (E) nuclear BUBR1, BUB3.1 and MAD2 colocalisation in yellow, (F–G) that BUBR1 and MAD2 interaction colocalised with (F) bright chromocenter spots stained with DAPI (blue channel) and with (G) the centromeric marker GFP-HTR12. n, nucleus. Bars, 25 µm (B, D and E), 5 µm (F and G).

In yeast and humans, BUBR1, BUB3 and MAD2 may be found together in large complexes (mitotic checkpoint complex) [7], [17], [19]. To carry out possible interactions between the cell cycle-regulated *A. thaliana* BUBR1, BUB3.1 and MAD2, a yeast two-hybrid split-ubiquitin approach was used. It is based on the fusion of the prey and the bait to the N- and C-terminal halves of ubiquitin (Nub and Cub, respectively), which are then able to form a native-like ubiquitin upon interaction [22]. Ubiquitin-specific proteases recognize the reconstituted ubiquitin and cleave off a reporter protein, URA3, linked to the C terminus of Cub and whose degradation results in uracil auxotrophy and 5-FOA resistance. Coexpression of BUBR1:Cub:URA3 with either Nub:BUB3.1 and Nub:MAD2 conferred resistance to 5-FOA, indicating that BUBR1 interacted with both BUB3.1 and MAD2. BUB3.1 and MAD2 also interacted (Fig. 2A). These interactions were confirmed in a reciprocal bait-prey experiment.

To better characterise the physical interactions between BUBR1, BUB3.1 and MAD2, we performed *in planta* localisation of these interactions. Following transient expression of the appropriate gene construct in *Nicotiana benthamiana* leaf epidermis, BUBR1 fused to GFP (BUBR1:GFP) was specifically targeted to the nucleus (*n* = 30; Fig. 2B), whereas BUB3.1:GFP and MAD2:GFP were detected in both the nucleus and the cytoplasm (*n* = 30; Fig. 2B). Using bimolecular fluorescence complementation (BiFC; [23], we demonstrated a close interaction between BUBR1, BUB3.1 and MAD2. Coexpression of the constructs encoding BUBR1:YC (BUBR1 fused to the C-terminal half of YFP) and BUB3.1:YN (BUB3 fused to the N-terminal half of YFP) resulted in the reconstituted YFP complexes only in the nuclei (*n* = 20; Fig. 2C–D). In addition, BUB3.1 interacted with MAD2 in the nuclei and cytoplasm of epidermal cells (*n* = 20; Fig. 2D). No YFP fluorescence was detected in negative control experiments in which BUBR1:YN, BUB3.1:YN, BUB3.1:YC or MAD2:YC was produced together with the corresponding vector control (*n* = 30). Coexpression of the constructs encoding BUB3.1:YC, MAD2:YN, and BUBR1:GFP showed that BUB3.1 and MAD2 interact, and that they co-localise with BUBR1 in the nucleus (*n* = 20; Fig. 2E). Interactions between BUBR1:YC and MAD2:YN were observed exclusively in the nucleus, as bright subnuclear foci (*n* = 40; Fig. 2F). Within the nuclei, fluorescence signals were localised with the core of bright DAPI-stained condensed chromocenters (Fig. 2F). Using the centromeric Histone H3 variant from *A. thaliana* GFP:HTR12 (CENH3, AT1G01370) as *in vivo* marker for centromeres [24]–[26], we confirmed that BUBR1 and MAD2 interact at interphase centromeres (*n* = 10; Fig. 2G) corresponding to the position on the chromosome at which kinetochore proteins associate.

### BUBR1, BUB3.1 and MAD2 localised to the kinetochores following SAC activation

In metazoan cells, the BUBR1, BUB3 and MAD2 proteins are specifically localised to the kinetochores following the activation of the SAC by global defects in spindle assembly in cells treated with microtubule poisons [20], [27]–[29]. The maize and wheat MAD2 proteins are the only plant SAC proteins for which localisation to unattached kinetochores has been demonstrated [11], [12]. By combined direct immunofluorescence of maize MAD2 and CENPC, the identity of the MAD2-positive regions as kinetochores has been demonstrated [11].

**Figure 3:**
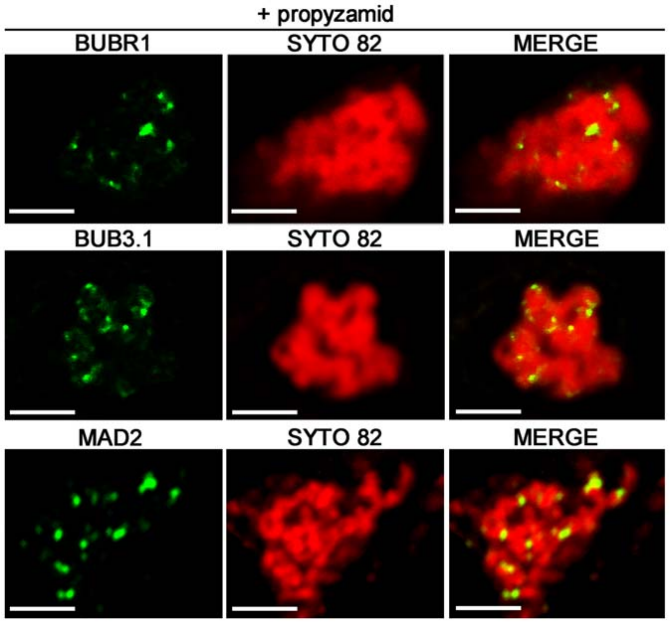
Subcellular distribution of BUBR1:GFP, BUB3.1:GFP and MAD2:GFP fusion proteins in propyzamid-treated tobacco cells. Single optical section of prometaphase-like arrested cells expressing BUBR1:GFP, BUB3.1:GFP and MAD2:GFP fusion constructs, 1 h after propyzamid treatment. In merged images, the yellow colour corresponds to BUBR1:GFP, BUB3.1:GFP or MAD2:GFP (green channel) colocalisation with SYTO 82 (red channel). BUBR1:GFP, BUB3.1:GFP and MAD2:GFP localise *in vivo* to the kinetochores of chromosomes stained with *SYTO 82* orange-fluorescent nucleic acid stain. Bars, 5 µm.

To gain insight into the spindle checkpoint activation in plant, we profiled the spatial distribution of *A. thaliana* SAC proteins in tobacco cell cultures stably expressing the BUBR1:GFP, BUB3.1:GFP and MAD2:GFP constructs. At a prometaphase-like stage, following treatment with the microtubule-destabilizing herbicide propyzamid, which prevents the formation of microtubule-kinetochore attachments, the MAD2 fusion protein was found to cluster strongly in bright spots on condensing chromosomes corresponding to unattached kinetochores (*n* = 20; Fig. 3). Similar localisation was observed for the BUB3.1 and BUBR1 fusion proteins (*n* = 20; Fig. 3). Thus, the plant BUBR1, BUB3.1 and MAD2 partners identified in this study are all in place at the unattached kinetochores and may therefore fulfil the evolutionarily conserved functions of SAC proteins, delaying anaphase until all the chromosomes are attached to both poles of the spindle.

**Figure 4:**
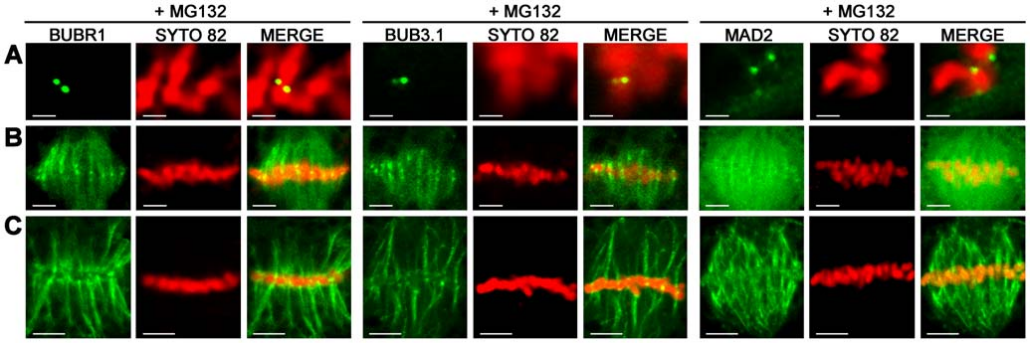
Subcellular localisation of BUBR1, BUB3.1 and MAD2 in MG132-treated tobacco cells. Single optical section of cells expressing BUBR1:GFP, BUB3.1:GFP and MAD2:GFP fusion constructs (green channel) after treatment with 100 µM MG132. Chromosomes in living cells were stained with SYTO 82 (red channel). In merged images, the yellow colour corresponds to the colocalisation of BUBR1:GFP, BUB3.1:GFP or MAD2:GFP with SYTO 82. (A) Two hours after MG132 treatment, BUBR1, BUB3.1 and MAD2 were localised into two bright spots per condensed chromosome, corresponding to kinetochores. (B) When chromosomes were arrested in metaphase, BUBR1, BUB3.1 and MAD2 localised to all the kinetochores of chromosomes arrested in metaphase. A diffuse signal in the metaphase spindle apparatus was also observed for BUBR1, BUB3.1 and MAD2. (C) Three hours after MG132 treatment, BUBR1, BUB3.1 and MAD2 localised to bright spots corresponding to the kinetochores of chromosomes and staining accumulated onto MT-like structures within the spindle in metaphase arrested cells. Bars, 2 µm (A), 5 µm (B and C).

We further analysed plant SAC protein distribution in living cells in cases of delayed anaphase onset. As anaphase initiation requires the ubiquitylation and degradation by the 26S proteasome of key mitotic regulators [2], such as the separase inhibitor securin and the Cdk1 subunit cyclin B, we studied cells that had been treated with the proteasome inhibitor MG132. The MG132 tripeptide has been shown to be a very efficient proteasome inhibitor in mammalian and plant cell cultures. It preserves metaphase spindles and kinetochore-microtubule (kMT) attachments but inhibits the onset of anaphase [30]. As previously reported in plants [30], [31], two hours after the addition of this molecule to a concentration of 100 µM, tobacco cells arrested in metaphase were found to have highly condensed chromosomes (*n* = 30; Fig. 4A). At this time point, *A. thaliana* BUBR1, BUB3.1 and MAD2 were localised to the sister kinetochores of condensed chromosomes in metaphase arrested cells (*n* = 20; Fig. 4A). In cells in which chromosomes were aligned at the spindle equator, BUBR1, BUB3.1 and MAD2 were found to be present in all the kinetochores (*n* = 15; Fig. 4B). Progressively, much of the BUBR1, BUB3.1, MAD2:GFP-derived fluorescence took on a fibrillar appearance, probably as a result of association with the acentrosomal metaphase spindle apparatus (*n* = 30; Fig. 4B). Three hours after MG132 treatment, the initially diffuse spindle BUBR1, BUB3.1 and MAD2:GFP staining accumulated progressively onto MT-like structures within the spindle (*n* = 25; Fig. 4C). At this time point, bright spots corresponding to kinetochores were also detected for BUBR1, BUB3.1 and MAD2 (*n* = 25; Fig 4C). To determine if the MT-like SAC fluorescence was in fact MT dependent, we treated BUBR1:GFP cells with the MT-stabilizing agents Paclitaxel. Three hours after MG132 treatment, the adjunction of Paclitaxel dramatically intensified the fibrillar nature of BUBR1:GFP (*n*>20; Fig. S4). In addition, immunostaining of β-tubulin confirmed that BUBR1 colocalized with spindle MTs when proteolysis is blocked by MG132 (*n*>10; Fig. S4). Previous reports have provided evidence for the motor-assisted transport of human MAD2 complexes from kinetochores to the spindle poles along MTs [32]. This mechanism may play an important role in removing checkpoint proteins from the kinetochores and turning off the checkpoint. Based on our observations, plant SAC proteins have an intriguing intracellular distribution, apparently accumulating onto both kinetochores and the spindle MTs in cell arrested in metaphase.

### SAC inactivation in normal cell division

**Figure 5:**
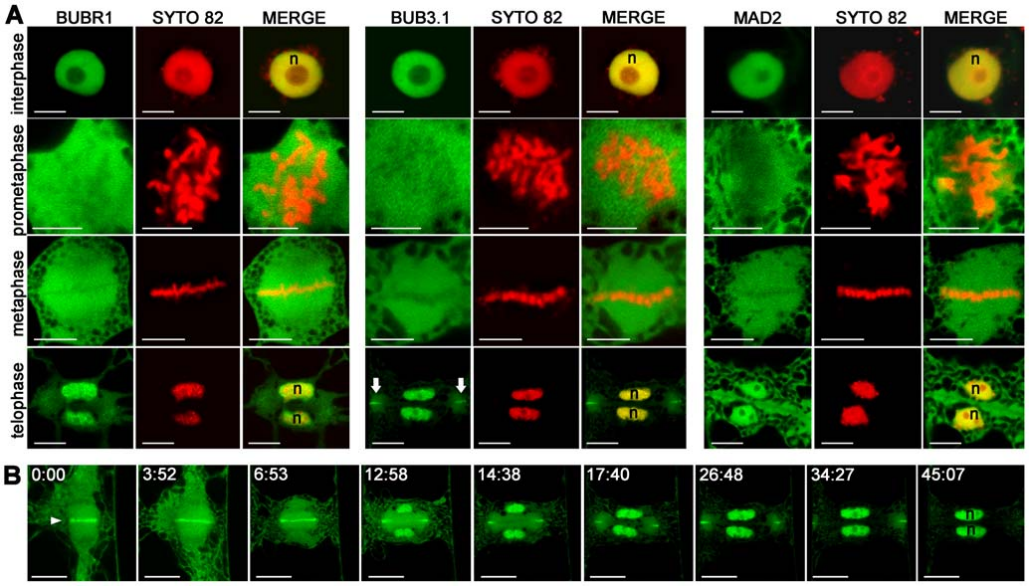
Subcellular localisation of BUBR1, BUB3.1 and MAD2 in tobacco cells undergoing normal mitosis. (A) Single optical section of cells expressing BUBR1:GFP, BUB3.1:GFP and MAD2:GFP fusion constructs (green channel). Chromosomes in living cells were stained with SYTO 82 (red channel). In merged images, the yellow colour corresponds to the colocalisation of BUBR1:GFP, BUB3.1:GFP or MAD2:GFP with SYTO 82. By telophase, BUB3.1:GFP was detected in daughter nuclei (n) and in the midline at the cell periphery (arrow), forming a ring around the edge of the newly formed cell plate. (B) Selected frames from a fluorescence time-lapse analysis of the distribution of BUB3.1:GFP during cytokinesis. Single optical section of a cell expressing the BUB3.1:GFP fusion construct (green channel). After chromosome separation, BUB3.1 is localised along the midline of the anaphase spindle (arrowhead). During telophase, BUB3.1 is gradually transferred into the daughter nuclei. During phragmoplast extension from the centre to the periphery of the cell, BUB3.1 localises with the margin of the expanded phragmoplast. At the end of telophase, BUB3.1 is present at the cell periphery, forming a ring around the edge of the newly formed cell plate. This specific localisation at the phragmoplast midline disappeared when the newly formed cell plate completely separated the two daughter cells. At the end of cytokinesis, BUB3.1 was again concentrated in the nucleus. Time is in min:s. Bars, 10 µm.

We then investigated the distribution of *A. thaliana* SAC proteins *in vivo* in normal mitosis conditions, when SAC is inactivated. This was made possible since the expression of the chimeric proteins did not prevent cell cycle progression. In the absence of SAC activation, BUBR1 was found exclusively in nuclei stained with SYTO 82 during interphase (*n* = 30; Fig. 5A). BUB3.1 and MAD2 proteins were localised to the nucleus and gave a weak cytoplasmic signal during interphase (*n* = 30; Fig. 5A). In early prophase, BUBR1, BUB3.1 and MAD2 became localised in the cytoplasm following nuclear envelope breakdown and remained there until the end of metaphase (*n* = 10; Fig. 5A). By telophase, when a new nuclear envelope forms around each set of separated sister chromosomes, *A. thaliana* BUBR1 and MAD2 were again concentrated in the nucleus (*n* = 15; Fig. 5A).

Overall, our data show that checkpoint proteins are only recruited at kinetochore in case of damage in spindle assembly. During normal mitosis, BUBR1, BUB3.1 and MAD2 staining at the kinetochore was not detected, inconsistent with reports for metazoan cells [2], [27], [33]. We propose that organism-specific differences in the behaviour of SAC are likely to reflect evolutionary divergence in the mechanics of spindle assembly rather than extensive differences in the pathways of checkpoint signalling. Animal cells undergo an open mitosis in which prometaphase chromosomes are initially free of spindle microtubules after nuclear envelope breakdown. High levels of MAD and BUB proteins are present on these unattached kinetochores [2], [27], [33]. Plant cells undergo mitosis in which acentrosomal pro-spindle assembly is initiated before nuclear envelope breakdown [34]. Our data suggest that in plant, kinetochores do not recruit high level of these SAC proteins during normal mitosis which is consistent with the idea that plant chromosomes are continuously linked to MTs.

### BUB3.1 localised to phragmoplast midline during plant cytokinesis

BUB3.1 displayed an unexpected distribution during cytokinesis in late anaphase to telophase in plant cells. It first appeared in the centre of the forming cell plate, and was subsequently redistributed to the growing margins of the cell plate as the cell plate grew outwards. Time-lapse analysis showed *A. thaliana* BUB3.1 was strongly localised to the anaphase spindle midline after chromosome separation in late anaphase (*n* = 15; Fig. 5B). During the final stages of cell division, a cytokinetic apparatus unique to plants, the phragmoplast, was generated. The phragmoplast directs Golgi-derived vesicles to the midline where they fuse to form a cell plate, permitting the separation of the daughter cells. During telophase, a strong BUB3.1:GFP signal was detected in the early phragmoplast midline and in the newly formed daughter nuclei (*n* = 17; Fig. 5B). At the end of telophase, when the phragmoplast was fully expanded, BUB3.1 was observed at the cell periphery, forming a ring around the edge of the newly formed cell plate (*n* = 20; Fig. 5A–B). This signal disappeared when the fully expanded cell plate completely separated the two daughter cells (*n* = 18; Fig. 5B). This BUB3.1 subcellular localisation appeared intriguing since metazoan and yeast BUB3 has not been described to be involved in cytokinesis. In animal cells, after sister chromosomes have separated, the remaining non-kinetochore MTs form a structure called the spindle midzone. The spindle midzone was compressed by the ingressing cleavage furrow. This spindle remnant also persists during cytokinesis in plant cells, where it becomes the early phragmoplast. The difference is that, instead of being the focus of constriction, as in animals, the central spindle/early phragmoplast opens out as a ring that directs Golgi-derived vesicles to the midline where they fuse to form a cell plate.

We found that, during outward cytokinesis, BUB3.1 was specifically localised to the phragmoplast midline, at which the cell plate was held by phragmoplast MTs. The BUBR1 and MAD2 proteins did not follow this pattern. Thus, BUB3.1, in addition to its known role in the spindle assembly checkpoint itself, may have a plant-specific role in late mitosis coordinating phragmoplast expansion. The phragmoplast midline defines the interface between phragmoplast MT plus-ends and the newly formed cell plate. Recent studies indicated that the phragmoplast midline could contain linker molecules that help to stabilize MT plus-ends and connect them to cell plate membranes. This results in optimally organized phragmoplast MTs that deliver the Golgi-derived vesicles to the growing cell plate [35].

We hypothesize, that BUB3.1 could be part of a MT plus-end capture complex associated with other “phragmoplast midline proteins” and then may regulate phragmoplast expansion, essential for cytokinesis. An analysis of the cell cycle regulators present in synchronised *A. thaliana* cell cultures showed that *BUB3.1* expression was coregulated with the expression of other cytokinesis-related genes [9]. In addition, the AURORA-like kinase 1 [36], the microtubule-associated protein MAP65-3 [37], the molecular motor kinesin PAKRP2 [38] and the CDC27/HOBBIT APC/C subunit [39] have phragmoplast midline distributions similar to that of BUB3.1 during cytokinesis. However, none of these proteins has been reported to be localised to both unattached kinetochores and the phragmoplast midline. The association of BUB3.1 with both these structures suggests that plants may coordinate spindle assembly and cytokinesis through shared machinery. This study provides clues to the possible functional links between the spindle and phragmoplast assembly checkpoints, ensuring failsafe mitosis.

## Materials and Methods

### Sequence identification and gene cloning

*A. thaliana* proteins orthologous to human BUB3, BUBR1 and MAD2 were identified by the OrthoMCL [13] clustering of six proteomes based on standard parameters. The six proteomes compared were those of *A. thaliana* (TAIR, http://www.arabidopsis.org), *Homo sapiens* (http://www.ncbi.nlm.nih.gov/projects/CCDS/CcdsBrowse.cgi), *Oryza sativa* (http://rice.plantbiology.msu.edu/), *Drosophila melanogaster* (http://flybase.org/), *Caenorhabditis elegans* (http://wormbase.org/) and *Meloidogyne incognita* (http://meloidogyne.toulouse.inra.fr/ [40]). Interpro scans (http://www.ebi.ac.uk/interpro) were used to study domain organisation. The *A. thaliana* BUBR1, BUB3.1, MAD2 and *HTR12*/*CENP-A* coding sequences were amplified by PCR, using specific primers (Fig. S5). They were inserted into the pDON207 donor vector and then into the pK7FWG2, or pK7WGF2 for *HTR12*, plant expression vector and BiFC vectors (pAM-35SS-GWY-YFPc and pAM-35SS-GWY-YFPn), using Gateway Technology (Invitrogen).

### Promoter analysis and histochemical localisation of GUS activity

For the promoter:GUS fusion, fragments of the 1365 bp, 1001 bp, 999 bp and 1000 bp immediately upstream from the start codon, for *BUB3.1*, *BUB3.2, MAD2* and *BUBR1*, respectively, were amplified by PCR (Fig. S5), inserted into the pDON207 donor vector and then into the pKGWFS7 plant vector, using Gateway Technology (Invitrogen). Wild-type (WS ecotype) *A. thaliana* plants were stably transformed and GUS activity was assayed histochemically, as previously described [37], on 10 independent transformed plants for each construct. Samples were observed with a Zeiss Axioplan 2 microscope and images analysed with AxioVision 4.7 (Zeiss).

### Yeast two-hybrid split-ubiquitin assay

The split-ubiquitin assay was carried out in *S. cerevisiae* strain JD53, as previously described [41]. The *BUBR1*, *BUB3.1* and *MAD2* coding sequences were inserted into the GW:Cub:URA3 bait vector (pMKZ) and the NuI:GW prey vector, using the Gateway system. Standard procedures were used for yeast growth and transformation. Transformants were selected on 5-fluoroorotic acid (5-FOA) plates containing minimal medium with yeast nitrogen base without amino acids (Difco) and glucose, supplemented with lysine, leucine, uracil (M-HW), and 1 mg/ml 5-FOA.

### *N. benthamiana* transformation and cell cultures

*N. benthamiana* plants were grown under continuous light for 1 month at 26°C. Infiltration of *Agrobacterium tumefaciens* into tobacco leaves was as described [42] and plants were analysed two days after infiltration. For tobacco cell culture establishment, *N. benthamiana* leaves were cocultured two days with *A. tumefaciens* in the dark at 26°C, rinsed in a liquid MS medium containing 3% sucrose and 150 mg/l cefotaxime (Sigma). The tissue was blotted dry and placed on regeneration medium (MS medium, 3% sucrose, 1.0 mg/l indole acetic acid, and 0.1 mg/l benzyladenine, Sigma, 0.8% agar), and supplemented with 150 mg/l cefotaxime and 50 mg/l kanamycin. Explants were incubated in a controlled growth chamber at 26°C. All explants were subcultured onto fresh regeneration/selection medium every 10 days. Two explants were used to generate suspension cultures: stably transformed explants were placed on MS medium supplemented with 0.5 mg/l 2,4D (2,4-dichlorophenoxyacetic acid) and 40 mg/l kanamycin for the induction of callus, which was transferred into liquid MS medium supplemented with 1 mg/l 2,4D and 50 mg/l kanamycin. The cultures were incubated at 26°C in the dark with continuous shaking.

### Drug treatments and microscopy

Optical sections of tobacco leaf epidermal cells or tobacco cell cultures were observed with a ×63 water immersion apochromatic objective (numerical aperture 1.2, Zeiss) fitted to an inverted confocal microscope (Axiovert 200 M, LSM510 META; Zeiss) at 25°C. GFP and SYTO 82 (Molecular Probes) fluorescence were monitored in Channel mode with a BP 505–530, 488 beam splitters and LP 530 filters for GFP and 545 nm beam splitters for SYTO 82 (488 nm excitation line). For DAPI staining, cells were first fixed in 1×PBS+2% paraformaldehyde in PBS (1 x) supplemented with 0.05% Triton X-100. DNA was stained *in vivo* with the orange fluorescent dye SYTO 82 (2 µM final concentration). Propyzamid (Sigma), Paclitaxel (Sigma) and carbobenzoxyl-leucinyl-leucinyl-leucinal (MG132; kindly provided by M. C. Criqui, IBMP, Strasbourg, France) were used at final concentrations of 50 µM, 50 µM and 100 µM, respectively. These preparations were stored for no more than one month at −20°C. The samples treated with MG132 were collected at different time point to be observed during metaphase arrest by *in vivo* confocal microscopy. For Propyzamid and Paclitaxel treatments, samples were collected 10 min after drug adjunction and used immediately for observation. Digital images were analysed using LSM Image Browser (Zeiss), imported to Photoshop CS2 (Adobe) and contrast/brightness was uniformly changed. For immunolocalization of β-tubulin, samples were collected 3 hours after MG132 treatment. Cells were first fixed in 1×PBS+2% paraformaldehyde supplemented with 0.05% Triton X-100. Immunolabeling was performed according to Ritzenthaler et al.[43]. Cells were incubated overnight with the monoclonal anti-β-tubulin clone TUB 2.1 (Sigma-Aldrich). Two hours incubation at room temperature was performed with Alexa 596 goat antimouse IgG (Molecular Probes, Eugene, OR, USA). DNA was stained with 1 µg.ml^−1^ 4′,6-diamidino-2-phenylindole (DAPI, Sigma) in PBS 1 x buffer. GFP and Alexa 596 (Molecular Probes) fluorescences were monitored in Channel mode with a BP 505–530, HFT 488 beam splitters for GFP and LP 530 filters NFT, 545 nm beam splitters for Alexa Red (488 nm excitation line).

## Supporting Information

Figure S1 Sequence comparison of BUBR1/MAD3-related proteins. (A) Domain organisation of Arabidopsis thaliana AtBUBR1, Homo sapiens HsBUBR1 and Saccharomyces cerevisiae ScMAD3. Proteins are drawn to scale. (B) Alignment of AtBUBR1 protein from A. thaliana (AtBUBR1, At2g33560) with MAD3 protein from Schizosaccharomyces pombe (SpMAD3, O59767) and the NH2-terminal domains of BUBR1/MAD3 proteins from human (HsBUBR1, O60566), Xenopus larvei (XBUB1B, Q8JGT8) and S. cerevisiae (ScMAD3, P47074). The MAD3-BUB1 domain (PF08311), KEN boxes and BUB3-binding domain of hBUB1B are indicated.
(0.04 MB PDF)

Figure S2 Sequence comparison of BUB3-related proteins. (A) Domain organisation of Arabidopsis thaliana AtBUB3.1 and Homo sapiens HsBUB3. (B) Alignment of the BUB3-related proteins from A. thaliana (AtBUB3.1, At3g19590; AtBUB3.2, At1g49910), Drosophila melanogaster (DmBUB3, NP477381), Homo sapiens (HsBUB3, O43684), Mus musculus (mBUB3, Q9WVA3), Xenopus larvei (XBUB3, Q98UH2) and Saccharomyces cerevisiae (ScBUB3, P26449). The WD-40 repeats are underlined and the BUB3 WD signature sequence indicated by asterisks.
(0.02 MB PDF)

Figure S3 Sequence comparison of MAD2-related proteins. (A) Domain organisation of Arabidopsis thaliana AtMAD2 and human HsMAD2. (B) Alignment of the MAD2-related proteins from A. thaliana (AtMAD2, At3g25980), Zea mays (ZmMAD2, Q9XFH3), mouse (mMAD2, Q5HZH8), human (HsMAD2, AAC50781), Xenopus larvei (XMAD2, AAB41527) and Saccharomyces cerevisiae (ScMAD2, P40958). The HORMA domain (PF02301) is underlined. Identical amino acid residues are coloured.
(0.02 MB PDF)

Figure S4 Subcellular localisation of BUBR1 in MG132-treated tobacco cells. (A) Single optical images of cells expressing BUBR1:GFP fusion construct (green channel) treated with 100 µM MG132 (3 h), and then with 50 µM paclitaxel (10 min). Chromosomes in living cells were stained with SYTO 82 (red channel). The adjunction of Paclitaxel dramatically intensified the spindle MT-like structures of BUBR1:GFP. (B) Co-visualisation of MT spindle apparatus and BUBR1, three hours after 100 µM MG132 treatments. In merged image, the yellow colour corresponds to BUBR1:GFP (green channel) colocalisation with β-tubulin immunostaining (red channel). Bars, 5 µm.
(0.28 MB PDF)

Figure S5 Gateway primers used in this study.
(0.01 MB PDF)
