# Supplementary material for: Spindle Assembly Checkpoint Protein Dynamics Reveal Conserved and Unsuspected Roles in Plant Cell Division
Source: PLoS One. 2009 Aug 27;4(8):e6757. doi: 10.1371/journal.pone.0006757 (PMC2728542; doi:10.1371/journal.pone.0006757)
Supplement: Figure S1 — Sequence comparison of BUBR1/MAD3-related proteins. (A) Domain organisation of Arabidopsis thaliana AtBUBR1, Homo sapiens HsBUBR1 and Saccharomyces cerevisiae ScMAD3. Proteins are drawn to scale. (B) Alignment of AtBUBR1 protein from A. thaliana (AtBUBR1, At2g33560) with MAD3 protein from Schizosaccharomyces pombe (SpMAD3, O59767) and the NH2-terminal domains of BUBR1/MAD3 proteins from human (HsBUBR1, O60566), Xenopus larvei (XBUB1B, Q8JGT8) and S. cerevisiae (ScMAD3, P47074). The MAD3-BUB1 domain (PF08311), KEN boxes and BUB3-binding domain of hBUB1B are indicated. (0.04 MB PDF) [file pone.0006757.s001.pdf]

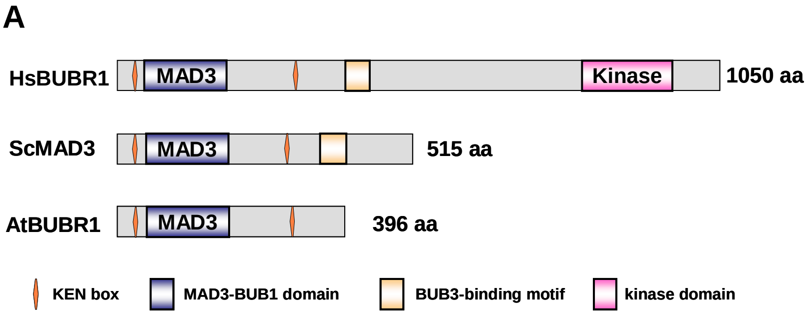

B

```
                                                         KEN
AtBUBR1        (1) MAAETKVQVSDPEAEFLNSKQETGYEWELFKENVRPLKRGRNVGILNHALKSHSDHQLRK
HsBUBR1        (1) -----MAAVKKEGGALSEAMSLEGDEWELSKENVQPLRQGRIMSTLQGALAQESACNNT-
XBUB1B         (1) -------------------MAQAGDEWELSKENVQPLRQGRVMSTLQEVLSQQESASHTA
ScMAD3         (1) -MKAYAKKRISYMPSSPSQNVINFEEIETQKENILPLKEGRSAAALSKAIHQPLVE----
SpMAD3         (1) -----------MEPLDAGKNWVHMDVIEQSKENIEPRKAGHSASALAKSSSRNHTEKEVA

                                         MAD3-BUB1 domain
AtBUBR1       (61) NLIE-KRRNLIEAIDEYEGDDPLSPWIECIKWVQEAFPPG--GECSGLLVIYEQCVRKFW
HsBUBR1       (56) LQQQ--KRAFEYEIRFYTGNDPLDVWDRYISWTEQNYPQG--GKESNMSTLLERAVEALQ
XBUB1B        (42) VQQQ--KQAFELELRFYAGDDPLDVWDRYIKWAEQAFPQG--GKESNLCPLLERGVKIFH
ScMAD3        (60) INQVKSSFEQRLIDELPALSDPITLYLEYIKWLNNAYPQGGNSKQSGMLTLLERCLSHLK
SpMAD3        (50) GLQKERMGHERKIETSESLDDPLQVWIDYIKWTLDNFPQGE-TKTSGLVTLLERCTREFV

AtBUBR1      (118) HSERYKDDLRYLKVWLEYAEHCA-----DAEVIYKFLEVNEIGKTHAVYYIAYALHIEFK
HsBUBR1      (111) GEKRYYSDPRFLNLWLKLGRLCN-----EPLDMYSYLHNQGIGVSLAQFYISWAEEYEAR
XBUB1B        (98) EEQRYYDDLRYLNICLKFANFCS-----EPLDLYSYLHSQGIGVSHSLLYITWAEQFEAR
ScMAD3       (116) DLERYRNDVRFLKIWFWYIELFTRNSFMESRDIFMYMLRNGIGSELASFYEEFTNLLIQK
SpMAD3       (109) RNPLYKDDYRYLRIWMQYVNYID-----EPVELFSFLAHHHIGQESSIFYEEYANYFESR

AtBUBR1      (173) NKVKTANEIFNLGISRDAKPVEKLNDAYKKFMVRTMRRSNTADEEPKENNDLPSRSFGTL
HsBUBR1      (166) ENFRKADAIFQEGIQQKAEPLERLQSQHRQFQARVSRQTLLALEKE-----------EEE
XBUB1B       (153) GNFKKADSMFQQGMQCKAEPLEKLEIHHRQFQARVSRQVLQGISEG-----------PDV
ScMAD3       (176) EKFQYAVKILQLGIKNKARPNKVLEDRLNHLLRELGENNIQLGNEIS---------MDSL
SpMAD3       (164) GLFQKADEVYQKGKRMKAKPFLRFQQKYQQFTHRWLEFAPQSFSSN-----------TNS

AtBUBR1      (233) LSRGDNNARRQALGSSNPQAKKLKPNQSSKTPFAIYADAVSDTTSGNQPESDKS------
HsBUBR1      (215) EVFESSVPQRSTLAELKSKGKKTARAPIIRVGGALKAPSQNRGLQNPFPQQMQNNSRITV
XBUB1B       (202) EEPELSEPQRSSLADLKSRGKTKAKVPVNRVGDSIKSRPQGLGLQAAPPQQIPNRSRFSV
SpMAD3       (213) VNPLQTTFESTNIQEIS---QSRTKISKPKFKFSVYSDADGSGKDGQ-------------
ScMAD3       (227) ESTVLGKTRSEFVNRLELANQNGTSSDVNLTKNNVFVDGEESDVELFETPN--R------

                                                    KEN
AtBUBR1      (287) ------------RPEFGSWLMLGGRAERNKENNSLPRKWASFKVPQK-----PIVRTVAA
HsBUBR1      (275) FDENADEASTAELSKPTVQPWIAPPMPRAKENELQAGPWNTGRSLEHRPRGNTASLIAVP
XBUB1B       (262) FDENAAMSAAQELPSLTPQQWTAPPPARSKENEQRARPWNSGRPSRN---GHQAPVSELP
ScMAD3       (279) ------------GVYRDGWENFDLKAERNKENNLRISLLEANTNLGE---------LKQH
SpMAD3       (257) ------------P---GTWQTLGTVDQRRKENNISATSWVGEKLPLK--------SPRKL

AtBUBR1      (330) ASASTFEVFVDEEECTEEEEEKKKNDETISSSSNVLPLNGGREIKKETELLRQNPLRHFP
HsBUBR1      (335) AVLPSFTPYVEETARQPVMTPCKIEPSINHILSTRKPGKEEGDPLQRVQSHQQASEEKKE
XBUB1B       (319) QSLPSFTPYVDEGAQHQTVTPCKINPAVTSVLSSRKPGK-DEDPLQRVQNNSQG---KEE
ScMAD3       (318) EMLSQKKRPYDEKLPIFRDSIGRSDPVYQMINTKDQ---------------------KPE
SpMAD3       (294) DPLGKFQVHCDEEVSKE-------------------------------------------

                             BUB3 binding domain
AtBUBR1      (390) PNSFLRR--------------------------------------(396/396)
HsBUBR1      (395) ------KMMYCKEKIYA-GVGEFSFEEIRAEVFRKKLKEQREALL(433/1050)
XBUB1B       (375) ------TVMYCKDKVYA-GVEEFSLEEIRAEIYMAKVRRKREDDLQ(413/1041)
ScMAD3       (357) KIDCNFKLIYCEDEESKGGRLEFSLEEVLAIS-RNVYKRVRTNRKH(401/515)
SpMAD3       (310) ---------------------------------------------(310/310)
```

Figure S1. Sequence comparison of BUBR1/MAD3-related proteins. (A) Domain organisation of *Arabidopsis thaliana* AtBUBR1, *Homo sapiens* HsBUBR1 and *Saccharomyces cerevisiae* ScMAD3. Proteins are drawn to scale. (B) Alignment of AtBUBR1 protein from *A. thaliana* (AtBUBR1, At2g33560) with MAD3 protein from *Schizosaccharomyces pombe* (SpMAD3, O59767) and the NH2-terminal domains of BUBR1/MAD3 proteins from human (HsBUBR1, O60566), *Xenopus larvei* (XBUB1B, Q8JGT8) and *S. cerevisiae* (ScMAD3, P47074). The MAD3-BUB1 domain (PF08311), KEN boxes and BUB3-binding domain of hBUB1B are indicated.
