# Supplementary material for: Spindle Assembly Checkpoint Protein Dynamics Reveal Conserved and Unsuspected Roles in Plant Cell Division
Source: PLoS One. 2009 Aug 27;4(8):e6757. doi: 10.1371/journal.pone.0006757 (PMC2728542; doi:10.1371/journal.pone.0006757)
Supplement: Figure S2 — Sequence comparison of BUB3-related proteins. (A) Domain organisation of Arabidopsis thaliana AtBUB3.1 and Homo sapiens HsBUB3. (B) Alignment of the BUB3-related proteins from A. thaliana (AtBUB3.1, At3g19590; AtBUB3.2, At1g49910), Drosophila melanogaster (DmBUB3, NP477381), Homo sapiens (HsBUB3, O43684), Mus musculus (mBUB3, Q9WVA3), Xenopus larvei (XBUB3, Q98UH2) and Saccharomyces cerevisiae (ScBUB3, P26449). The WD-40 repeats are underlined and the BUB3 WD signature sequence indicated by asterisks. (0.02 MB PDF) [file pone.0006757.s002.pdf]

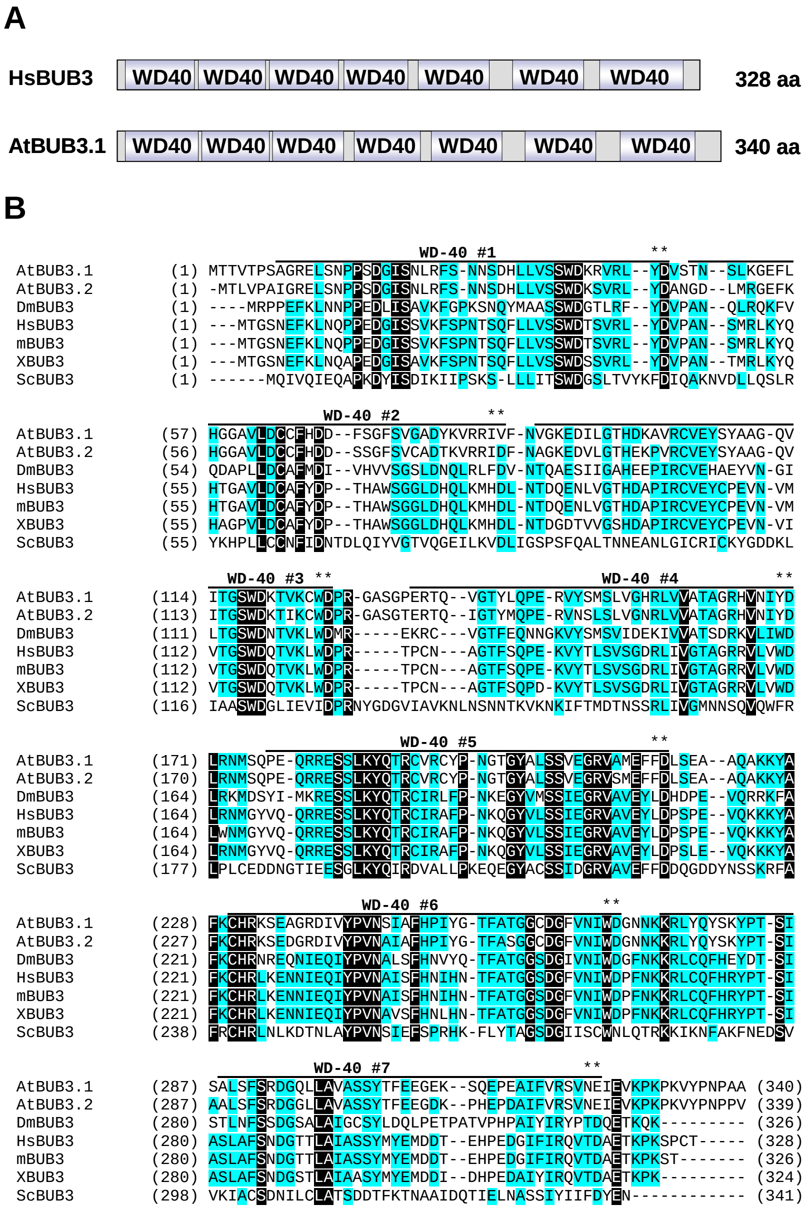

Figure S2. Sequence comparison of BUB3-related proteins. (A) Domain organisation of *Arabidopsis thaliana* AtBUB3.1 and *Homo sapiens* HsBUB3. (B) Alignment of the BUB3-related proteins from *A. thaliana* (AtBUB3.1, At3g19590; AtBUB3.2, At1g49910), *Drosophila melanogaster* (DmBUB3, NP477381), *Homo sapiens* (HsBUB3, O43684), *Mus musculus* (mBUB3, Q9WVA3), *Xenopus larvei* (XBUB3, Q98UH2) and *Saccharomyces cerevisiae* (ScBUB3, P26449). The WD-40 repeats are underlined and the BUB3 WD signature sequence indicated by asterisks.
