# Supplementary material for: Spindle Assembly Checkpoint Protein Dynamics Reveal Conserved and Unsuspected Roles in Plant Cell Division
Source: PLoS One. 2009 Aug 27;4(8):e6757. doi: 10.1371/journal.pone.0006757 (PMC2728542; doi:10.1371/journal.pone.0006757)
Supplement: Figure S3 — Sequence comparison of MAD2-related proteins. (A) Domain organisation of Arabidopsis thaliana AtMAD2 and human HsMAD2. (B) Alignment of the MAD2-related proteins from A. thaliana (AtMAD2, At3g25980), Zea mays (ZmMAD2, Q9XFH3), mouse (mMAD2, Q5HZH8), human (HsMAD2, AAC50781), Xenopus larvei (XMAD2, AAB41527) and Saccharomyces cerevisiae (ScMAD2, P40958). The HORMA domain (PF02301) is underlined. Identical amino acid residues are coloured. (0.02 MB PDF) [file pone.0006757.s003.pdf]

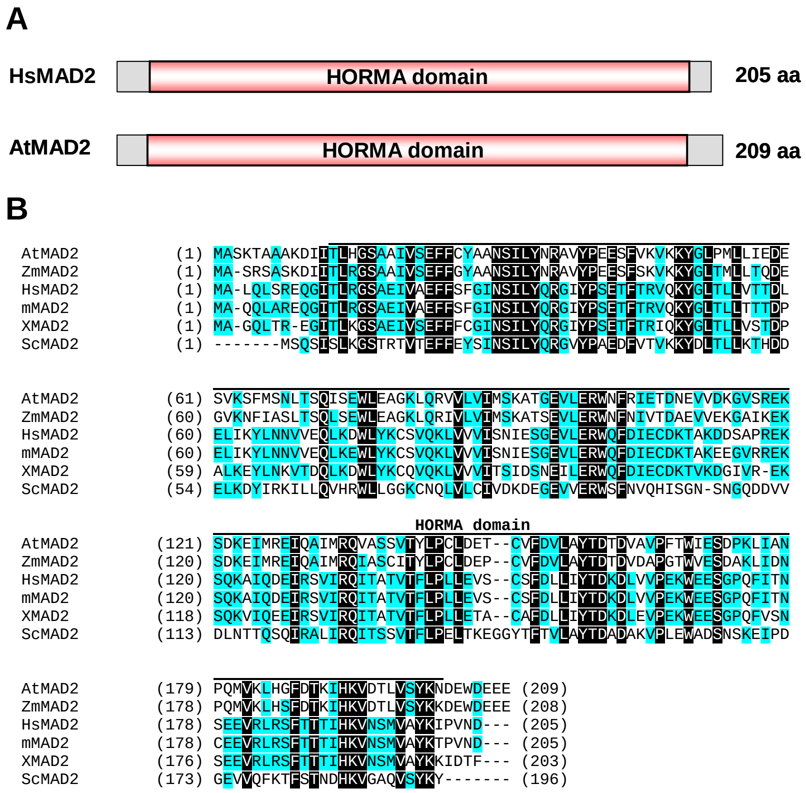

Figure S3. Sequence comparison of MAD2-related proteins. (A) Domain organisation of *Arabidopsis thaliana* AtMAD2 and human HsMAD2. (B) Alignment of the MAD2-related proteins from *A. thaliana* (AtMAD2, At3g25980), *Zea mays* (ZmMAD2, Q9XFH3), mouse (mMAD2, Q5HZH8), human (HsMAD2, AAC50781), *Xenopus larvei* (XMAD2, AAB41527) and *Saccharomyces cerevisiae* (ScMAD2, P40958). The HORMA domain (PF02301) is underlined. Identical amino acid residues are colored.
