# Supplementary material for: Spindle Assembly Checkpoint Protein Dynamics Reveal Conserved and Unsuspected Roles in Plant Cell Division
Source: PLoS One. 2009 Aug 27;4(8):e6757. doi: 10.1371/journal.pone.0006757 (PMC2728542; doi:10.1371/journal.pone.0006757)
Supplement: Figure S4 — Subcellular localisation of BUBR1 in MG132-treated tobacco cells. (A) Single optical images of cells expressing BUBR1:GFP fusion construct (green channel) treated with 100 µM MG132 (3 h), and then with 50 µM paclitaxel (10 min). Chromosomes in living cells were stained with SYTO 82 (red channel). The adjunction of Paclitaxel dramatically intensified the spindle MT-like structures of BUBR1:GFP. (B) Co-visualisation of MT spindle apparatus and BUBR1, three hours after 100 µM MG132 treatments. In merged image, the yellow colour corresponds to BUBR1:GFP (green channel) colocalisation with β-tubulin immunostaining (red channel). Bars, 5 µm. (0.28 MB PDF) [file pone.0006757.s004.pdf]

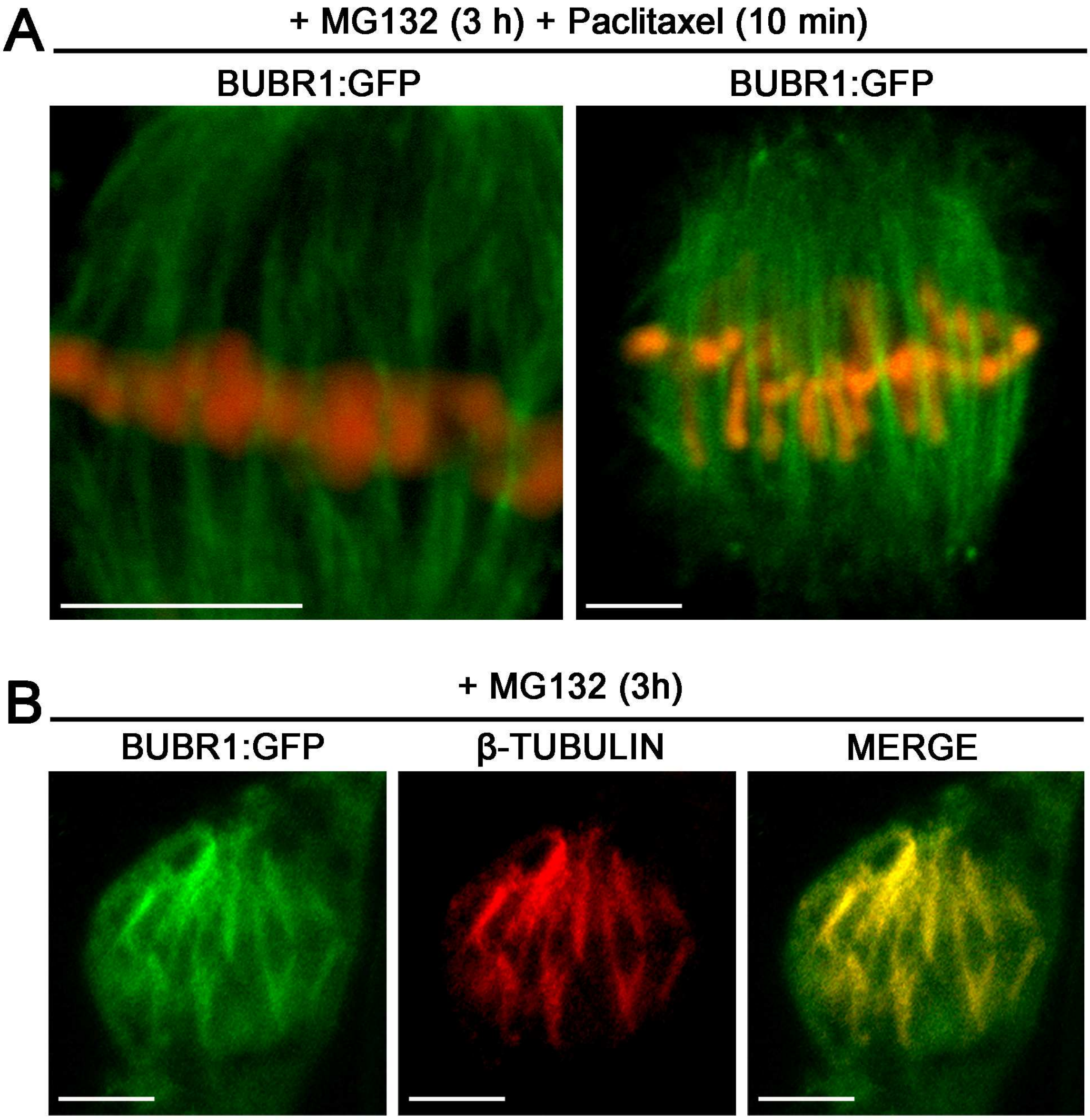

Figure S4. Subcellular localisation of BUBR1 in MG132-treated tobacco cells.

(A) Single optical images of cells expressing BUBR1:GFP fusion construct (green channel) treated with 100 µM MG132 (3 h) and then with 50 µM paclitaxel (10 min). Chromosomes in living cells were stained with SYTO 82 (red channel). The adjunction of Paclitaxel dramatically intensified the spindle MT-like structures of BUBR1:GFP. (B) Co-visualisation of MT spindle apparatus and BUBR1, three hours after 100 µM MG132 treatments. In merged image, the yellow colour corresponds to BUBR1:GFP (green channel) colocalisation with β-tubulin immunostaining (red channel). Bars, 5 µm.
