# Supplementary material for: Spindle Assembly Checkpoint Protein Dynamics Reveal Conserved and Unsuspected Roles in Plant Cell Division
Source: PLoS One. 2009 Aug 27;4(8):e6757. doi: 10.1371/journal.pone.0006757 (PMC2728542; doi:10.1371/journal.pone.0006757)
Supplement: Figure S5 — Gateway primers used in this study. (0.01 MB PDF) [file pone.0006757.s005.pdf]

| Gene (AGI) | Primer name | Primer sequence (5'-3') |
|---|---|---|
| **AtBUB3.1 (At3g19590)** | AtBUB3.1_Promoter 5' | AAAAAGCAGGCTTCGGCAGATGTGTTTTTAAGTCGG |
| | AtBUB3.1_Promoter 3' | AGAAAGCTGGGTGTTTTCCCGTTGAAGATCACA |
| | AtBUB3.1_E1_ATG | AAAAAGCAGGCTTCACC**ATG**ACGACTGTGACTCCGTCC |
| | AtBUB3.1_E1_noATG | AAAAAGCAGGCTTCACGACTGTGACTCCGTCC |
| | AtBUB3.1_E9R_STOP | AGAAAGCTGGGTG**TCA**CGCCGCAGGATTCGGGTATA |
| | AtBUB3.1_E9R_noSTOP | AGAAAGCTGGGTGTCCCGCCGCAGGATTCGGGTATA |
| **AtBUB3.2 (At1g49910)** | AtBUB3.2_Promoter 5' | AAAAAGCAGGCTTCGATGTGTGTTGTGTGAGTTACA |
| | AtBUB3.2_Promoter 3' | AGAAAGCTGGGTGCCTGGAAAATACGAATTTTTAG |
| **AtBUBR1 (At2g33560)** | AtBUBR1_Promoter 5' | AAAAAGCAGGCTTCATGGAATTCTTAAACGTACGGC |
| | AtBUBR1_Promoter 3' | AGAAAGCTGGGTGCGTCGTTTCTTCGAGCAAAT |
| | AtBUBR1_E1_ATG | AAAAAGCAGGCTTCACC**ATG**GCAGCCGAAACGAAGGT |
| | AtBUBR1_E1_noATG | AAAAAGCAGGCTTCACCGCAGCCGAAACGAAGGT |
| | AtBUBR1_E11R_STOP | AGAAAGCTGGGTG**TCA**TCGTAGGAAGCTGTTGGGTGGGAAA |
| | AtBUBR1_E11R_noSTOP | AGAAAGCTGGGTGTCTTCGTAGGAAGCTGTTGGGTGGGAAA |
| **AtMAD2 (At3g25980)** | AtMAD2_Promoter 5' | AAAAAGCAGGCTTCTCTGTGTTCATATGATGTCAAC |
| | AtMAD2_Promoter 3' | AGAAAGCTGGGTGGAGTCTAAGGATCGGTGATTT |
| | AtMAD2_E1_ATG | AAAAAGCAGGCTTCACC**ATG**GCGTCCAAAACAGCGGCTGCTA |
| | AtMAD2_E1_noATG | AAAAAGCAGGCTTCGCGTCCAAAACAGCGGCTGCTA |
| | AtMAD2_E7R_STOP | AGAAAGCTGGGTG**TCA**TTACTCTTCTTCATCCCACTCGTCG |
| | AtMAD2_E7R_noSTOP | AGAAAGCTGGGTGTTACTCTTCTTCATCCCACTCGTCG |
| **AtHTR12 (AT1G01370)** | AtHTR12_E1_ATG | AAAAAGCAGGCTTC**ATG**GCGAGAACCAAGCATCGC |
| | AtHTR12_E3_STOP | AGAAAGCTGGGTG**TCA**CCATGGTCTGCCTTTTCCTCC |

**Figure S5. Gateway primers used in this study.**
